# Gradual acquisition of immunity to severe malaria with increasing exposure

**DOI:** 10.1098/rspb.2014.2657

**Published:** 2015-02-22

**Authors:** Jamie T. Griffin, T. Déirdre Hollingsworth, Hugh Reyburn, Chris J. Drakeley, Eleanor M. Riley, Azra C. Ghani

**Affiliations:** 1 MRC Centre for Outbreak Analysis and Modelling, Department of Infectious Disease Epidemiology, Faculty of Medicine, Imperial College London, London W2 1PG, UK; 2 Mathematics Institute, University of Warwick, Coventry CV4 7AL, UK; 3 School of Life Sciences, University of Warwick, Coventry CV4 7AL, UK; 4 Department of Clinical Sciences, Liverpool School of Tropical Medicine, Pembroke Place, Liverpool L3 5QA, UK; 5 Faculty of Infectious and Tropical Diseases, London School of Hygiene and Tropical Medicine, Keppel Street, London WC1E 7HT, UK

**Keywords:** malaria, severe, *Plasmodium falciparum*, immunity, mathematical model

## Abstract

Previous analyses have suggested that immunity to non-cerebral severe malaria due to *Plasmodium falciparum* is acquired after only a few infections, whereas longitudinal studies show that some children experience multiple episodes of severe disease, suggesting that immunity may not be acquired so quickly. We fitted a mathematical model for the acquisition and loss of immunity to severe disease to the age distribution of severe malaria cases stratified by symptoms from a range of transmission settings in Tanzania, combined with data from several African countries on the age distribution and overall incidence of severe malaria. We found that immunity to severe disease was acquired more gradually with exposure than previously thought. The model also suggests that physiological changes, rather than exposure, may alter the symptoms of disease with increasing age, suggesting that a later age at infection would be associated with a higher proportion of cases presenting with cerebral malaria regardless of exposure. This has consequences for the expected pattern of severe disease as transmission changes. Careful monitoring of the decline in immunity associated with reduced transmission will therefore be needed to ensure rebound epidemics of severe and fatal malaria are avoided.

## 1. Background

A small minority of *Plasmodium falciparum* infections result in severe malaria, requiring admission to hospital [1]. Immunity to severe malaria is known to develop with repeat infections of *P. falciparum*, but the rate of acquisition of immunity is a subject of on-going study [2]. Manifestations of severe malaria have been shown to vary with age and transmission setting [3–9], and in response to changing transmission [10], suggesting that both age and exposure affect the pathophysiology of the disease. Previous analysis suggested that immunity to non-cerebral severe malaria is acquired after only one or two infections [11]. However, evidence from longitudinal studies shows that some young children experience multiple episodes of severe disease [12,13], suggesting that immunity may be acquired more gradually. Age patterns of disease are further confounded by variation in exposure to *P. falciparum* and immune responses, resulting in a high degree in variability in the number of disease episodes among children living in the same area [1,14].

A shift in the burden of severe malaria from younger to older ages with decreasing malaria transmission intensity is also associated with changes in the dominant manifestations of disease. In high transmission settings, severe malaria is concentrated among young children over the age of six months who most commonly present with severe malarial anaemia. At intermediate and low transmission settings, cases present in older children and adults with an increasing proportion of cerebral malaria [4,6,9,15–17], which is associated with higher case fatality ratios [4,18–22]. Despite this, the higher case fatality ratio is more than offset by the much lower incidence of severe malaria in low transmission settings, such that—overall—malaria-related mortality decreases with decreasing transmission [10]. Thus, although there have been concerns that malaria control may be accompanied by a paradoxical increase in mortality when transitioning from very high to intermediate levels of transmission [3,23–28], in practice, this has not been observed in two areas where there have been declines in transmission [10,29], perhaps due to the rapidity of the declines to low levels of transmission in these areas. However, a recent increase in malaria hospital admissions in western Kenya could be an example of this phenomenon [30], and so it remains an open question.

Few studies have quantified the rate of development of immunity to the different manifestations of severe malaria. Quantifying these processes is important to allow the prediction of the likely impact of interventions which are designed to reduce exposure during early years, including seasonal malaria chemoprophylaxis (SMC), use of insecticide-treated nets (ITNs) and vaccination via the Expanded Programme for Immunization (EPI), but which may leave older individuals at risk. A better understanding of the rate at which immunity is acquired is additionally likely to promote a better understanding of the pathophysiology of severe malaria.

In 1999, Gupta *et al.* used a mathematical model fitted to the age distribution of cerebral and non-cerebral malaria incidence across different transmission settings in Kenya and Gambia to estimate the rate of acquisition of immunity to these syndromes [11,31]. This model assumed that infections were acquired at a constant rate from birth, but that in young infants the proportion of cases with severe disease was reduced by passive immunity from maternal antibodies and that the proportion of cases with severe disease varied with exposure. Their analysis suggested that immunity to non-cerebral malaria was acquired after only a few infectious bites. However, in order to replicate the age distribution of non-cerebral malaria, alternative models were required that explicitly accounted for the exposure to multiple antigenic types of malaria, and increasing virulence of later infections [31]. The apparent contradiction between these two analyses—one suggesting rapid acquisition of immunity to severe disease and the other suggesting increasing case fatality with increasing age—has yet to be resolved. Indeed, there are still many unanswered questions about the development of immunity to severe malaria [2,32].

In an attempt to resolve some of these contradictions, we estimate the rate of acquisition of immunity to different forms of severe malaria by extending a published mathematical model of malaria transmission [33,34] to incorporate the development of immunity to severe disease. The model is fitted to data from Tanzania in which cases are stratified according to the main symptoms of severe disease—severe malarial anaemia, cerebral malaria and respiratory distress [4]—as well as to a wider summary analysis of severe disease patterns across different transmission intensities [5,35].

## 2. Material and methods

### (a) Tanzanian study

Full details of the Tanzanian study are published elsewhere [4] but are summarized briefly here. Data from hospital admissions for severe malaria from northeastern Tanzania were collected prospectively from nine hospitals over a period of one year from February 2002, and from one hospital for a period of six months from August 2002.

The population in this area is culturally and ethnically homogeneous, but exposed to highly variable levels of *P. falciparum* transmission intensity, which depend on the altitude from sea level to above 1800 m [36]. Children living in low-lying areas have much higher admission rates for severe malaria than children living in high-altitude villages [4]. In total, data from 1989 people with documented evidence of severe malaria (as defined in [4]) and known place of residence were included in this study, of whom 134 (6.7%) had a fatal outcome.

Severe malarial anaemia was defined as *P. falciparum* parasitaemia (of any density) with haemoglobin concentration (Hb) less than 5 g dl^−1^ (HaemoCue AB, Ängelholm, Sweden). Cerebral malaria was defined as *P. falciparum* parasitaemia (of any density) with Blantyre coma score (BCS) less than 4 and, if there was reduced response to pain, blood glucose level greater than 38 mg dl^−1^ (2.1 mmol l^−1^), no convulsions within 1 h of diagnosis and no anticonvulsants administered within 6 h of diagnosis. Malaria with respiratory distress was defined as parasitaemia (of any density) with lower chest wall inspiratory recession or abnormally deep respiration. Patients who did not meet any of these criteria but who met the criteria for inclusion in the study reported in [4] are classified as ‘other severe malaria’ cases.

Each ward was classified into one of six groups of differing transmission intensity: below 600 m, 600–1200 m and more than 1200 m.a.s.l. in each of Kilimanjaro and Tanga regions. The median journey time to the hospital that patients reported was also used in the analysis.

### (b) Other African data

To add information from areas with intermediate transmission, which were not represented in the Tanzanian study, we also used data on incidence of severe malaria from nine sites across Africa collected together by Marsh & Snow [35], together with data on the age distribution of severe malaria in each of these sites and the parasite prevalence in 2–10-year-olds reported by Okiro *et al.* [5]. In these studies, severe malaria was defined as a hospitalized case with a positive blood smear for *P. falciparum* where no other detectable cause for the clinical presentation could be identified. Some of the data were summarized in [3], in which the incidence of acute respiratory infection (ARI) was also reported using the same method for calculating the denominator population. The ARI incidence was similar between locations, suggesting that the calculated denominator was in a similar ratio to the true catchment population in the different sites.

### (c) Model structure and estimation of parameters

We used a published transmission model [33], which has recently been updated by fitting to extensive clinical disease data [34]. The model is described in full in the latter. In brief, the previously published model incorporates individuals ageing and being continuously exposed to infection and acquiring immunity. The rate of acquisition of this immunity depends on the level of exposure, as measured by the entomological inoculation rate (EIR). There are three effects of immunity in this previously published model: (i) a reduction in the probability of initial infection, with the most marked reductions in teenagers and adults, reflecting pre-erythrocytic immunity, but with only modest effect; (ii) a gradual reduction in the probability of developing clinical disease given infection, representing the development of blood-stage immunity over repeated exposures; and (iii) a lower parasite density in asymptomatic infection following repeated exposure, leading to a reduction in both the probability of detection and in onward infectiousness to mosquitoes. The model is stratified by age and by degree of exposure to mosquitoes. The model was validated by fitting to age-stratified data on the incidence of clinical disease, PCR positivity and parasite prevalence, and EIR measurements.

Here, we extended the model by adding another immunity function to capture the peak incidence of severe disease at younger ages than the peak incidence of uncomplicated malaria. As individuals are repeatedly infected with malaria, they acquire this immunity, measured by the variable *I*_VA_. At birth, individuals have some level of maternally derived immunity, *I*_VM_, which is also determined by exposure of mothers. Mathematically, this can be written as the level of this immunity in individuals of age *a* in an area with EIR *ɛ*, increasing with exposure as
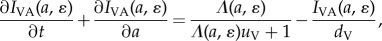
where *Λ*(*a*,*ɛ*) is the force of infection at age *a*, *u*_V_ represents a period during which immunity cannot be boosted following a previous boost and *d*_V_ governs the duration of immunity, representing the assumption that this immunity would decline in the absence of exposure. Maternal immunity *I*_VM_ is assumed to be at birth a proportion *P*_VM_ of the acquired immunity of a 20-year-old in the same transmission setting and to decay exponentially with rate 1/*d*_VM_.

The proportion of new infections that develop into severe disease declines as this level of immunity increases, with a sigmoidal function given by
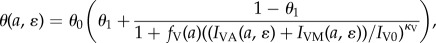
where *θ*_0_ is the proportion in naive individuals, *θ*_0_*θ*_1_ is the proportion in a maximally immune individual, and *I*_V,0_ and *κ*_V_ are scale and shape parameters. *f*_V_(*a*) is a purely age-dependent function that allows the effect of immunity to differ by age, given by *f*_V_(*a*) = 1−(1−*f*_V0_)/(1+(*a*/*a*_V_)*^*γ*_v_^*) at age *a*, with parameters *f*_V0_, *a*_V_ and *γ*_V_. Hence the incidence of severe malaria is *θ*(*a*,*ɛ*)*Λ*(*a*,*ɛ*).

The parameters allow for a flexible functional form for the dependence of the proportion *θ* on either exposure or age. The other model parameters not related to severe disease were fixed at the values estimated by Griffin *et al*. [34].

For each incident case of severe malaria the proportion of severe cases presenting with any individual symptom (severe anaemia, cerebral malaria or respiratory distress) was assumed to be age-dependent. The proportion of severe cases presenting with each symptom was assumed to be independent of the others and therefore any combination of the three symptoms occurs with a probability given by the product of the individual symptoms.

The proportion of severe malaria cases presenting with each symptom at age *a* is
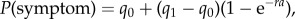
with a separate set of parameters *q*_0_, *q*_1_ and *r* for each symptom. The case fatality ratio was estimated for each combination of symptoms, with no age dependence (other than in the proportion of cases presenting with each symptom).

We included a journey-time-dependent probability of cases arriving in hospital as a number of studies have demonstrated that longer journey times to hospital are associated with a lower probability of arrival at a healthcare facility [37–39].

The differences in definition of the population at risk in the Tanzania study compared with the pooled summary studies from elsewhere in Africa give rise to markedly different scales for the overall incidence of disease for a similar age profile of disease (i.e. a similar transmission setting as defined by parasite prevalence), and we accounted for these differences by introducing scaling factors (see electronic supplementary material for further details).

The model was fitted to the data on incidence of different types of severe disease by age. The EIR in each setting was primarily informed by the parasite prevalence in that setting as the modelled relationship between EIR and parasite prevalence had previously been validated against a large number of settings [33,34]. Further details of the model-fitting methodology and the parameter estimates are given in the electronic supplementary material, text S1.

## 3. Results

**Figure 1.**
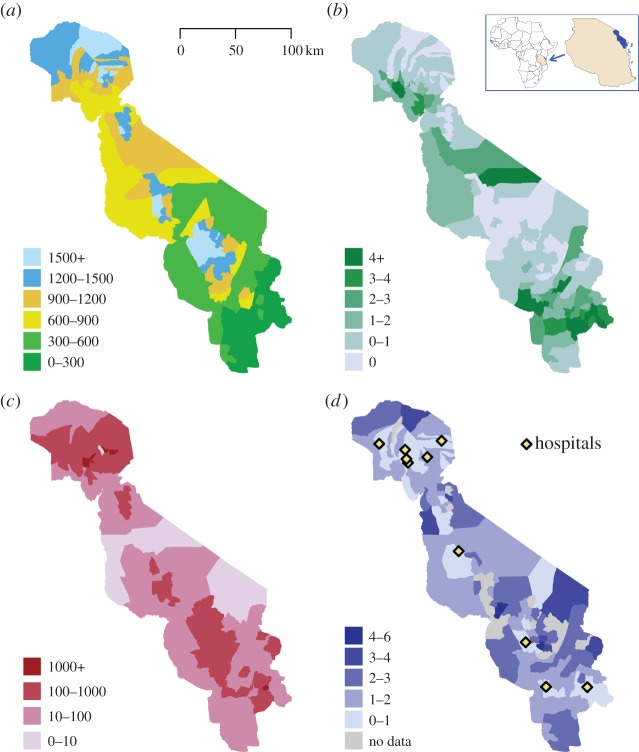
Geographical characteristics of study area, severe disease incidence, population density and travel time to hospital by ward. The study was conducted in northeastern Tanzania, where (*a*) the altitude increases from the coast in the southeast to the mountains in the northwest of the study area (altitude of each ward in metres). There is also geographical variation in (*b*) the incidence of severe malaria according to admissions to hospital per 1000 head of population, (*c*) population density per km^2^ and (*d*) median travel time to the hospital where the case was admitted by ward, in hours, as reported by patients' families (with the hospitals in the study marked with yellow diamonds).

People in the Tanzanian study lived at a wide range of altitudes, leading to a high degree of variability in malaria risk (figure 1*a*), with hospitals placed in areas of highest population density (figure 1*cd*). By far the highest incidence of severe malaria was seen in the low-altitude area in the southwest, near the sea, in Tanga (figure 1*b*). Just under half (47%) of the severe malaria cases, and 78% of cases who died, presented with at least one of cerebral malaria, severe malarial anaemia or respiratory distress, and 8% of cases were admitted with more than one of these syndromes. The combination of respiratory distress and anaemia accounted for 60% of all cases with more than one syndrome. In the highest transmission area (Tanga, altitude below 600 m), the majority of admissions were in the youngest age groups (43% aged below 1 year), the most common manifestation was severe malarial anaemia (47% of cases) and 3% of cases presented with cerebral malaria.

**Figure 2.**
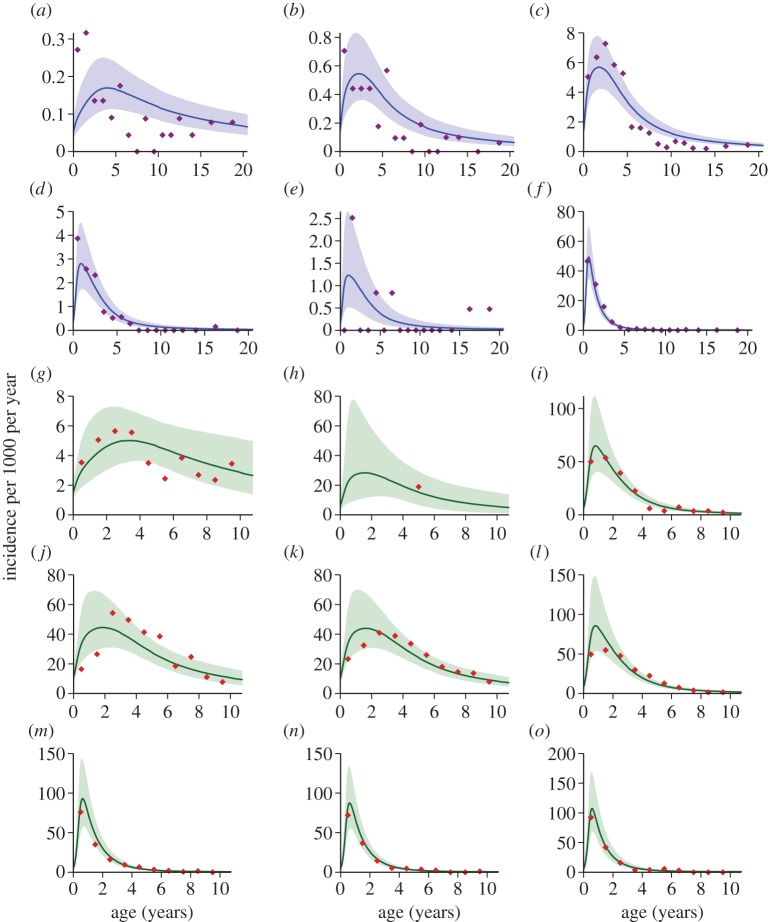
Age distribution of cases admitted to hospital for severe malaria by setting. The observed incidence of severe malaria by age alongside the model fit. The first two rows are data from Tanzania in order of increasing parasite prevalence: (*a*) Kilimanjaro high altitude, (*b*) Tanga high altitude, (*c*) Kilimanjaro mid-altitude, (*d*) Tanga mid-altitude, (*e*) Kilimanjaro low altitude and (*f*) Tanga low altitude. The last three rows are data from datasets presented by Okiro *et al*. [5] and Marsh & Snow [35], ordered by increasing parasite prevalence: (*g*) Bakau, Gambia; (*h*) Kilifi Township, Kenya; (*i*) Mponda, Malawi; (*j*) Foni Kansala, Gambia; (*k*) Sukuta, Gambia; (*l*) Kilifi North, Kenya; (*m*) Siaya, Kenya; (*n*) Kilifi South, Kenya; and (*o*) Ifakara, Tanzania. The shaded areas represent the 95% credible intervals of the fit. These are the raw data, without the scaling for different case definitions and catchment areas in these two groups of studies.

**Figure 3.**
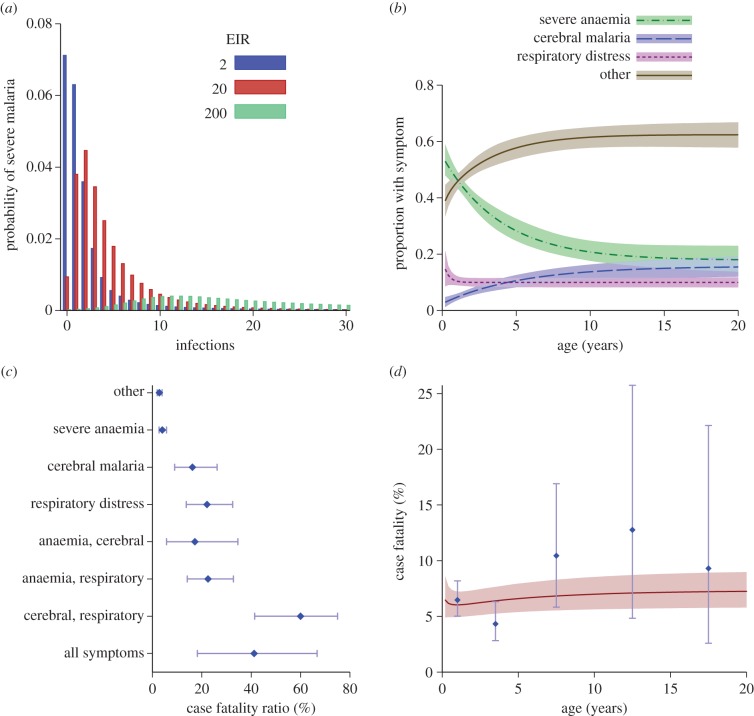
Model functions estimated by fitting to the data. (*a*) Proportion of infections that progress to severe malaria plotted against the number of infections experienced so far, for three different EIRs. (*b*) Proportion of cases of severe malaria of a given age that will present with severe anaemia, cerebral malaria, respiratory distress or none of these symptoms. (*c*) The case fatality ratio for severe malaria in this cohort depending on the combination of symptoms (point estimates, dots; 95% confidence intervals, bars). (*d*) Given a case of severe disease, the age-dependent symptoms (*b*) combined with the symptom-related mortality (*c*) can be combined to give a case fatality ratio by age (solid line), which can be compared with the observed data (blue). For (*b*) and (*d*), the lines represent the best fit and the shaded areas represent the 95% credible intervals. In (*b*) and (*c*), ‘other’ refers to those with severe malaria as defined in [4], but without severe anaemia, cerebral malaria or respiratory distress.

The best-fitting mathematical model captured the differences in admission patterns by age (figure 2) across the wide range of settings. As was previously described by Okiro *et al.* [5], these data show the age shift in severe malaria from younger to older ages as the transmission intensity decreases. Figure 3*a* shows the estimated proportion of infections that develop into severe malaria, plotted against the expected number of infections experienced so far. Overall, the proportion of infections resulting in severe disease is very low. In high transmission settings this proportion rises at first (due to loss of maternal immunity), before gradually falling. At an EIR of two infectious bites per person per year, the proportion of infections resulting in severe disease falls more rapidly, presumably because of age-related gains in immune system competence (i.e. at low EIRs, children age more between each infection), but still takes several infections to fall below 1/10 of the initial proportion. The presentation of different symptoms was estimated to be modified by age, with the proportion of cases presenting with severe anaemia declining from 55% (95% CI: 49–62) at birth to 21% (18–25) at age 10 years (figure 3*b*). The proportion of cases presenting with cerebral malaria increased from 2% (0.5–4) at birth to 14% (11–16) by age 10 years. The proportion of cases presenting with respiratory distress was approximately constant after the first year of life at 10%, consistent with previous analyses of these data, which found that the variable most closely associated with respiratory distress was travel time to hospital [4]. As expected, among children admitted with severe malaria, the case fatality ratio increased with the number of syndromes present and was higher for cases of cerebral malaria than for cases of severe malarial anaemia (figure 3*c*). The combination of age-related symptoms and symptom-related mortality gives an overall case fatality ratio of the fitted model between 6% and 7%, increasing gradually with age, although there is some indication that the fitted value underestimates the case fatality ratio at older ages (figure 3*d*). As shown in electronic supplementary material, figure S3, the assumption that the occurrence of each symptom depends only on age may not be valid as there were some differences in symptoms seen between two areas of differing transmission intensity.

**Figure 4.**
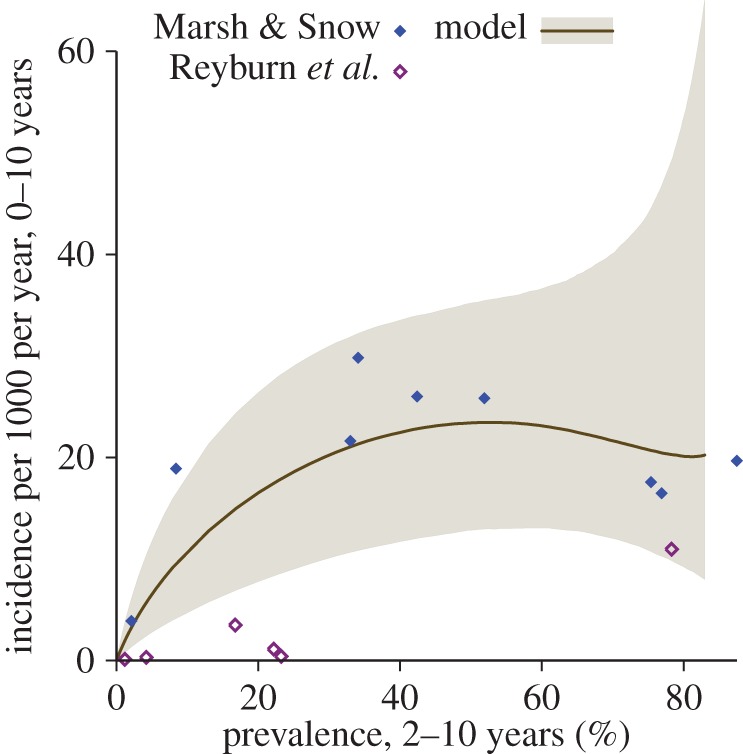
Changing admission patterns with increasing transmission. Incidence of severe malaria in 0–10-year-olds per 1000 population per year as a function of parasite prevalence in 2–10-year-olds. The Tanzanian data are shown without the scaling for different case definitions and denominator calculations. The line is the best fit of the model and the shaded area is the 95% credible interval. The points are the data published by Marsh & Snow [35] and by Reyburn *et al*. [4]. (Online version in colour.)

In the fitted mathematical model, the incidence of severe malaria increases with parasite prevalence, reaching a plateau when the prevalence in 2–10-year-olds is approximately 50% (figure 4). A slight decrease in incidence at very high prevalence was estimated but the uncertainty means that a continuing plateau or slight increase cannot be ruled out.

**Figure 5.**
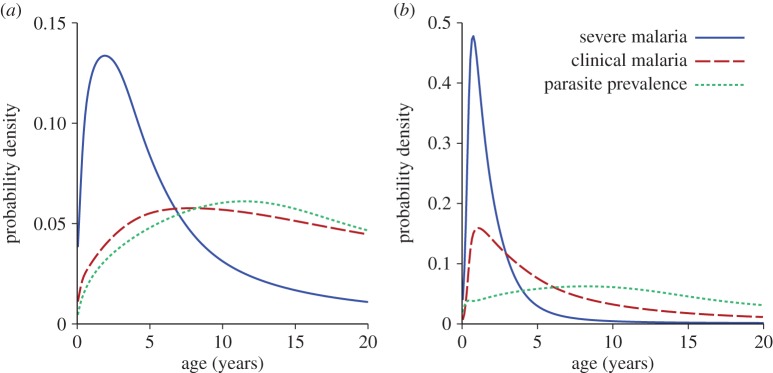
Age patterns of disease and infection. Model-predicted age distribution of incidence of severe malaria, clinical malaria and prevalence of parasites as detected by microscopy, at annual EIRs of (*a*) 2 and (*b*) 50. These EIRs correspond to parasite prevalences of 13% and 58%, respectively, in 2–10-year-olds. Each curve is the probability distribution of one outcome over 0–20-year-olds. (Online version in colour.)

Figure 5 shows the model-predicted age patterns of parasite prevalence, incidence of clinical malaria and incidence of severe malaria. As has previously been described [8], the model predicts a more rapid acquisition of immunity to severe malaria, followed by development of immunity to clinical malaria and finally in older children/adults to parasitaemia.

## 4. Discussion

Our analysis demonstrates that variations in patterns of severe malaria with age and transmission intensity can be explained by exposure- and age-driven acquisition of immunity, which determine the incidence of severe malaria, coupled with age-dependent physiological changes, which determine the dominant clinical syndrome of severe malaria. In accordance with previous studies [11,31], our analysis shows an increase in immunity with cumulative exposure. However, by including multiple syndromes and fitting a model across multiple transmission intensities simultaneously we estimate a more gradual acquisition of immunity to severe disease than previously suggested. While the overall *risk* of severe disease varies with force of infection, we were able to explain *patterns* of severe disease syndromes using a model of a solely physiological, age-dependent process. In particular, the proportion of the first few infections that lead to severe disease is highly dependent on the EIR (figure 3*a*). In a high-transmission setting, the first 30 infections are experienced very rapidly, and each individual infection has a low probability of progressing to severe disease episodes as initially infants are protected by maternal immunity, and then immunity is acquired rapidly. In a lower-transmission setting (e.g. EIR = 2), infections are experienced more slowly, and therefore maternal immunity is less important, but age-related gains in immune system competence decrease the proportion of infections that result in severe malaria. This interaction between acquisition of immunity to severe disease and age is a complex dynamic process, and our model provides a mechanism for investigating how disease patterns may change as transmission reduces.

In addition to the dynamics of severe disease overall, we have modelled the effect of age on symptoms. The proportion of severe cases presenting with severe malarial anaemia is estimated to decline steadily with age, whereas the proportion of cases presenting with cerebral malaria increases, and the proportion of cases presenting with respiratory distress was estimated to be independent of age. These observations suggest that the observed variation in severe disease presentation with age across a wide range of transmission settings may be driven by a combination of variation in the rate of acquisition of immunity to severe disease *per se* with exposure and a declining proportion of severe cases developing severe malarial anaemia with age. The increased proportion of severe cases developing cerebral malaria with increasing age, although real, is likely to have a smaller effect on the overall pattern of disease. The balance between acquired immunity and exposure results in an increasing incidence of severe malaria with increasing EIR, which appears to tail off slightly at very high EIR, as suggested by Snow *et al.* [3]. This combined modelling approach also allows us to show the shift in the burden of parasitaemia, clinical disease and severe disease with age in different transmission settings, as discussed by Marsh & Kinyanjui [40] and Langhorne *et al*. [2], showing the different age shifts in each type of infection (figure 5).

There are, of course, limitations to the use of hospital admission data for studies of severe illness. First, whether people with severe malaria come to the hospital at all, and how severely ill they are when they arrive, will be affected by access, both spatial and economic, and treatment-seeking behaviour [38]. Hospital data may thus not be representative of patterns of disease in the community, and in particular, may under-represent disease incidence and presentation in infants and very young children who are less able to attract attention to their illness [41,42]. Hospital data may also misrepresent the true incidence of different disease syndromes. For example, severe anaemia has a slower and less dramatic presentation than cerebral malaria, which may be more likely to cause death before effective treatment can be obtained, and this may result in biased estimates of relationships with age and exposure. In addition, the BCS, used to define cerebral malaria, may underestimate the incidence of this syndrome in infants, particularly below eight months of age, as they have not yet fully developed the ability to localize pain [43].

Furthermore, previous or subsequent episodes of mild or severe disease were not recorded for these cases. Ideally we would test our model predictions in longitudinal studies incorporating severe and uncomplicated malaria and asymptomatic exposure into the model framework. However, the resulting close monitoring and active case detection of malaria are likely to result in prompt and effective treatment of non-severe malaria such that severe malaria becomes less likely.

It should also be stressed that the majority of these data come from a small number of sites within a small number of countries. Studies such as this should be repeated in many other countries across Africa before the model can be reliably used to predict the patterns and absolute burden of severe malaria elsewhere. Factors that could affect the patterns of severe disease include nutritional status, socio-economic status, human and parasite genetics and seasonality. Furthermore, health systems vary widely across Africa, as does access to ACTs, which will affect the incidence of severe disease [44].

There are also limitations to this modelling approach. We have assumed that immunity to severe disease is acquired as a direct consequence of exposure but do not consider the biological mechanisms by which this immunity is acquired. Strain-specific immunity is one mechanism that can reproduce the observed relationship between age and disease risk [45–47]. However, antibody- and T-cell-mediated immunity to conserved antigens that is gradually acquired with exposure could also reproduce these risk relationships. Moreover, age-related changes in the predominant mechanisms of immunity (e.g. the balance between humoral and cellular mechanisms or between inflammatory and regulatory responses [48]) may explain the age-dependent changes in the proportions of different disease syndromes. Uncovering the biological mechanisms conferring both susceptibility and resistance to severe disease will aid future vaccine design as well as improve our understanding of the immune correlates of protection.

Given the progressive declines in transmission that have been observed in many settings in the last decade, it is likely that both the incidence and pattern of severe disease will continue to change in the coming years. If transmission continues to decline, reduced acquisition of immunity in young children coupled with gradual loss of immunity in older children and adults will leave a larger proportion of the population susceptible to severe disease. Although the absolute risk of severe disease—at any age—is expected to decline and remain low as long as transmission continues to decline, a very large at-risk population will emerge that is highly vulnerable should malaria transmission start to rise again. This at-risk population will include a large proportion of older children and adults, and this analysis suggests that severe disease is likely to present as cerebral malaria, with an associated high case fatality ratio. Careful monitoring of the decline in immunity associated with reduced transmission will therefore be needed to ensure rebound epidemics of severe and fatal malaria are avoided.

## Supplementary Material

Supplementary information

## Supplementary Material

Supplementary data

## Data accessibility

The data used in the analysis are contained in the online supplementary information.
