# Supplementary material for: Gradual acquisition of immunity to severe malaria with increasing exposure
Source: Proc Biol Sci. 2015 Feb 22;282(1801):20142657. doi: 10.1098/rspb.2014.2657 (PMC4309004; doi:10.1098/rspb.2014.2657)
Supplement: Supplementary information [file rspb20142657supp1.pdf]

Gradual acquisition of immunity to severe malaria with increasing exposure

Jamie T Griffin[1*†], T Déirdre Hollingsworth[2,3,4*], Hugh Reyburn[5], Chris J Drakeley[5], Eleanor M Riley[5], Azra C Ghani[1]

[1]MRC Centre for Outbreak Analysis and Modelling, Department of Infectious Disease Epidemiology, Faculty of Medicine, Imperial College London,

[2]Mathematics Institute, University of Warwick

[3]School of Life Sciences, University of Warwick

[4]Department of Clinical Sciences, Liverpool School of Tropical Medicine

[5]Faculty of Infectious & Tropical Diseases, London School of Hygiene and Tropical Medicine

[*] These authors contributed equally to the study.

[†] Corresponding author

Supplementary Information

Areas included in the analysis of individual-level data

The hospitals where data were collected for the study reported in [1] were in the Kilimanjaro and Tanga regions of North-East Tanzania. The study hospitals in Kilimanjaro region spanned the main population centres (see Figure 1 of the main text), and so we included all of this region in the analysis. In Tanga region, Tanga is a large town, none of whose hospitals were included in the original study. Also, none of the study hospitals were in the south-west Tanga region. Hence we excluded from the analysis areas around Tanga town and in the south and west of Tanga region. The included areas in Tanga region were Korogwe and Lushoto districts and parts of Muheza and Handeni districts. We excluded the remainder of Muheza and Handeni districts, plus Tanga, Pangani and Kilindi districts (Figure S1).

The wards included in Muheza and Handeni districts were: Bwembwera, Daluni, Kicheba, Kigongoi, Kilulu, Kisiwani, Kwafungo, Lusanga, Magila, Magoroto, Majengo, Maramba, Masuguru, Mbaramo, Mhinduro, Misalai, Misozwe, Mkuzi, Mtindiro, Ngomeni, Nkumba, Pande, Potwe, Songa, Tingeni and Zirai wards in Muheza district; and Kabuku, Kwamatuku, Kwedizinga, Mgambo and Segera wards in Handeni district.

Figure S1 Areas included in the analysis

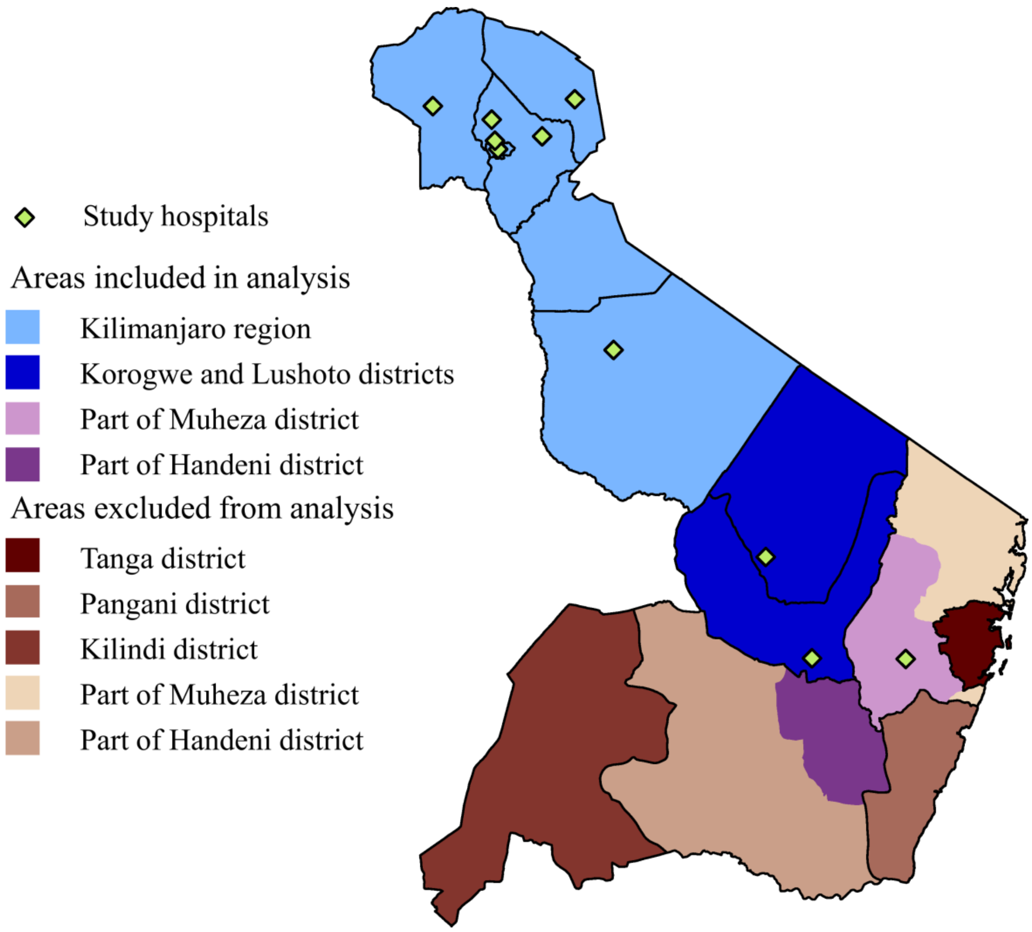

The ward of each person presenting at hospital was recorded, and so we were able to include in the analysis only those severe malaria patients who lived in the included wards, as well as using the populations of the included wards from the Tanzania National Census (2002) as the denominator for calculating incidence. The population at risk used to estimate incidence was considered to be the population of the wards from which cases were reported plus a handful of wards which were close to the hospital but reported no cases in the study period. At the district hospital in the highest transmission setting, children were recruited only every second day, due to the very large number of admissions. The data were weighted accordingly.

Model fitting methodology

Symptoms and mortality parameters

The parameters that determine the probability of experiencing each symptom, given that an episode of severe malaria occurs, and the case fatality ratio for each combination of symptoms were assumed to be independent of transmission intensity and past exposure. These parameters were estimated by maximum likelihood using the individual-level data from Tanzania.

Transmission model

The model-fitting for estimating the parameters relating to immunity against severe disease was done using Bayesian methods. The procedure for fitting the model to data was as follows:

- find the equilibrium model solution for each location, conditional on a given EIR, using the model and parameters from [2];
- for each location, find the predicted incidence of severe malaria by age;
- calculate the likelihood that the model produced the observed data.

We grouped the individual level data from Tanzania into six areas, and treated each as a single transmission setting for the purpose of calculating the model prediction: Tanga and Kilimanjaro regions were each divided into low, mid and high altitude (below 600m, 600-1200m and above 1200m respectively). Wards were classified according to the mean altitude, weighted by the population in each square kilometre. In the description that follows, these data are referred to as dataset A.

We additionally used data from nine sites across Africa reported in [3, 4], referred to as dataset B. The severe disease incidence data from Bakau and Sukuta, The Gambia, Kilifi

North, Siaya and Kilifi South, Kenya were originally published in [5], while the incidence data from Kilifi Township, Kenya, Mponda, Malawi, Foni Kansala, The Gambia and Ifakara, Tanzania were first published in [3]. The data point in [3] from an area of Ethiopia where malaria is epidemic rather than endemic was excluded since our analysis focusses on endemic malaria transmission. The denominator population for calculating incidence was taken as “pre-defined communities located with 15km of essential clinical services” ([3], figure 4).

The data from dataset B was grouped into 10 one year age groups, from birth to age 10. To model variation in incidence not explained by transmission intensity, we included a random effect u for each of the nine sites, having a normal distribution with standard deviation σ_V. For a given set of parameters let the model-predicted incidence of severe malaria in site j and age group i be μ_{ji}, and the person-time at risk and number of episodes be T_{ji} and y_{ji} respectively. We then assume that conditional on u, y_{ji} follows a negative binomial distribution with mean $e^u T_{ji}\mu_{ji}$ and dispersion parameter α_V. The random effects were integrated out of the likelihood by Gauss-Hermite quadrature.

In dataset B, where the population at risk was defined within a fixed radius of the hospital, the incidence rate was much higher than in dataset A, where the population of a larger area was taken as the population at risk. It is likely that there were also differences in health care availability leading to different catchment areas and different local population densities. These differences could not be resolved given the historic nature of the data. Instead of having random effects we therefore introduced scaling factors so that we could jointly fit the model to the two different types of data. We introduced a total of six scaling factors one for each of the transmission settings for the Tanzanian data. While this scaling was used to adjust overall incidence, it did not affect estimates of the age-profiles which were the main motivation for this analysis.

Dataset A was available at individual level. For each of the six areas into which the data were categorised, denote the scaling factor by r_k for area k. Then there was an additional scaling factor for each ward

$$s_j = \frac{1}{1+\left(x_j / x_0\right)^{\delta}}$$

where x_j is the median travel time to hospital reported by patients living in ward j, and x_0 and δ are parameters to be estimated. A small number of wards had no reported travel time. For these, the time was interpolated from neighbouring wards.

The data in each ward were put into 31 age groups. Starting at age 0, they were: 4 of width 3 months, 14 of width 1 year, and 13 of width 5 years. If the model-predicted incidence of severe malaria in ward j and age group i is μ_{ji}, and the person-time at risk is T_{ji}, then the expected number of events is

$$\upsilon_{ji} = r_{k(j)} s_j T_{ji} \mu_{ji}$$

where $k(j)$ is the area that ward j belongs to. In order to give comparable statistical weight to that given to the data in dataset B, we assume that there is a gamma-distributed random effect η for each of 14 age groups, 10 groups up to age 10 (as for dataset B), and then 10-15, 15-20, 20-40 and above 40 years, with each $\eta \sim Gamma\left(\alpha_V, 1/\alpha_V\right)$ being common to all wards. Then conditional on η, the number of severe disease episodes follows a Poisson distribution with mean $\eta\upsilon_{ji}$. The random effects η can be analytically integrated out of the likelihood [6].

The parasite prevalence in 2 to 10 year-olds was also known for each site in dataset B. This was modelled using a beta-binomial likelihood with site-level normally distributed random effects for the logit prevalence, as in [2].

For each site, we fitted the EIR as parameters, with log-normal prior distributions. The log EIR for each site in dataset B was given a prior mean based on [7], and a standard deviation of 2, so that the prior distribution is relatively flat and information on the transmission intensity comes mainly from the parasite prevalence. The fitting to uncomplicated malaria, parasite prevalence and EIR data reported in [2] included parasite prevalence data from 24 villages from the Kilimanjaro and Tanga regions, which for model fitting were grouped into the same six areas by region and altitude as dataset A here. Hence we had an estimate of the log EIR for each area, which we took as the prior mean, and we took the standard deviation to be 0.3.

Parameter estimates

Tables S1 and S2 contain the maximum likelihood estimates and 95% confidence intervals for the parameters determining which symptoms occur and the case fatality of each combination of symptoms. These were estimated from dataset A.

Table S1 Parameters determining probability of each symptom occurring. The parameters are defined in the main text.

	Maximum likelihood estimates and 95% confidence intervals		
Symptom	q_0	q_1	r
Severe anaemia	0.548 (0.490, 0.620)	0.178 (0.125, 0.229)	0.252 (0.141, 0.434)
Cerebral malaria	0.024 (0.005, 0.045)	0.158 (0.122, 0.222)	0.189 (0.076, 0.329)
Respiratory distress	0.185 (0.045, 1.000)	0.100 (0.082, 0.118)	2.970 (0.417, 47.3)

Table S2 Case fatality ratio (%) for each combination of symptoms with 95% confidence intervals

Symptoms	Estimate	95% CI
None	2.8	1.9, 3.9
Severe anaemia	4.0	2.7, 5.8
Cerebral malaria	16.2	9.2, 25.8
Respiratory distress	22.1	13.9, 32.3
Anaemia and cerebral	17.2	6.9, 33.7
Anaemia and respiratory	22.5	14.4, 32.5
Cerebral and respiratory	60.0	43.5, 74.9
All symptoms	41.2	20.7, 64.4

All transmission model parameters not related to severe disease were fixed at the values estimated in [2]. Table S3 lists the prior values and poster estimates and credible intervals of the severe disease parameters which were fitted here.

Table S3 Transmission model parameters related to severe disease

Parameter description	Symbol	Prior median and 95% interval	Posterior median and 95% credible interval
Acquired immunity			
Probability with no immunity	θ_0	0.5 (0.025, 0.975)	0.0749 (0.0302, 0.150)
Maximum relative reduction	θ_1	0.5 (0.025, 0.975)	1.19E^{-4} (3.02E^{-5}, 4.42E^{-4})
Inverse of decay rate	d_V	Fixed at 30 years	
Scale parameter	I_{V0}	0.0085 (0.000022, 4.94)	1.05 (0.57, 1.92)
Shape parameter	κ_V	2.01 (0.88, 3.06)	1.99 (1.55, 2.56)
Duration in which immunity is not boosted	u_V	7 days (0.28, 178)	11.3 days (0.76, 53.6)
Maternal immunity			
New-born immunity relative to mother's	P_{VM}	0.5 (0.025, 0.975)	0.197 (0.122, 0.460)
Inverse of decay rate of maternal immunity	d_{VM}	194 days (96.1, 342)	76.6 days (52.2, 111)
Travel kernel for dataset A			
Time-scale	x_0	2.0 hours (0, 36.6)	2.63 hours (2.52, 2.74)
Shape parameter	δ	1.82 (0.07, 7.79)	6.07 (5.18, 7.11)
Scaling factors r for recorded severe disease incidence in dataset A<br>By region and altitude			
Kilimanjaro: <600m		1.0 (0.02, 50)	0.056 (0.022, 0.144)
600-1200m		" "	0.286 (0.164, 0.641)
>1200m		" "	0.032 (0.016, 0.082)
Tanga: <600m		" "	0.617 (0.327, 1.19)
600-1200m		" "	0.084 (0.047, 0.181)
>1200m		" "	0.049 (0.026, 0.117)

Additional graphs of results

Travel time to hospital

Figure S2a shows the distribution of travel times to hospital in dataset A for all severe disease patients included in the analysis for whom this data was available, up to 10 hours. Each bar includes the upper limit: for example reported times of exactly 1 hour are included in the first bar, not the second bar. Most patients (79%) travelled less than two hours to hospital (47% 1 hour or less, 32% 1 to 2 hours), but a substantial number travelled for many hours, with the longest journey times being 10, 12 and 20 hours.

We estimate that the proportion of severe malaria patients reaching hospital decreases sharply as their travel time to hospital increases from two to four hours (Figure S2b), which is consistent with previously published work reviewed in [8]. This suggests that the overall incidence of severe malaria and malaria mortality in this study could be an underestimate due to the high probability of missing cases amongst populations more than 2 hours away.

Figure S2 (a) Reported travel times to hospital among severe disease patients. (b) Probability that a case of severe malaria will present to hospital by travel time to the hospital for that ward in hours, relative to someone with zero travel time.

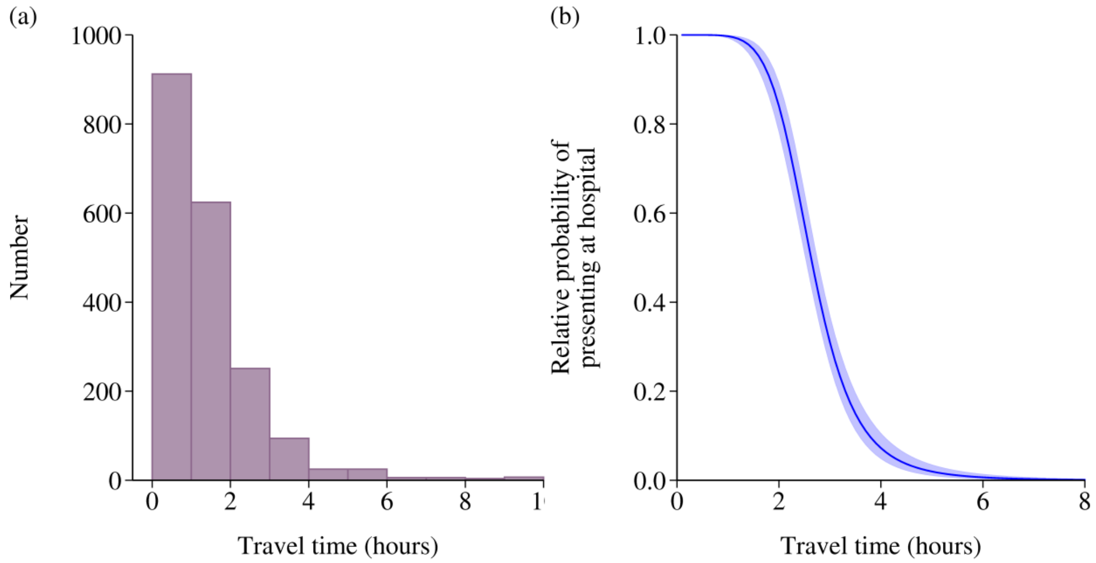

Observed and fitted symptoms by age

We assume in the fitted model that the probability of each type of symptom given that there is an episode of severe malaria is independent of transmission intensity, depending only on age. Figure S3 shows the observed probability of each of the three symptoms, or of none of

them in the whole Tanzanian dataset and also in two subsets of the data, low altitude Tanga and mid altitude Kilimanjaro: 89% of the patients in the analysis were from these areas. The former has much higher transmission than the latter. For plotting, the data are aggregated into the age groups 0-2, 2-5, 5-10 and 10-20 years.

Generally the model fits the overall dataset well, although at older ages the model may slightly under-estimate the probability of developing respiratory distress. However, in each age group the proportion of severe cases with severe anaemia is lower in mid altitude Kilimanjaro than in low altitude Tanga, and the proportion with none of the three symptoms and the proportion with cerebral malaria are both higher.

Figure S3 Observed probability of each symptom among those meeting the criteria for severe malaria in [1], with 95% binomial confidence intervals, and fitted model.

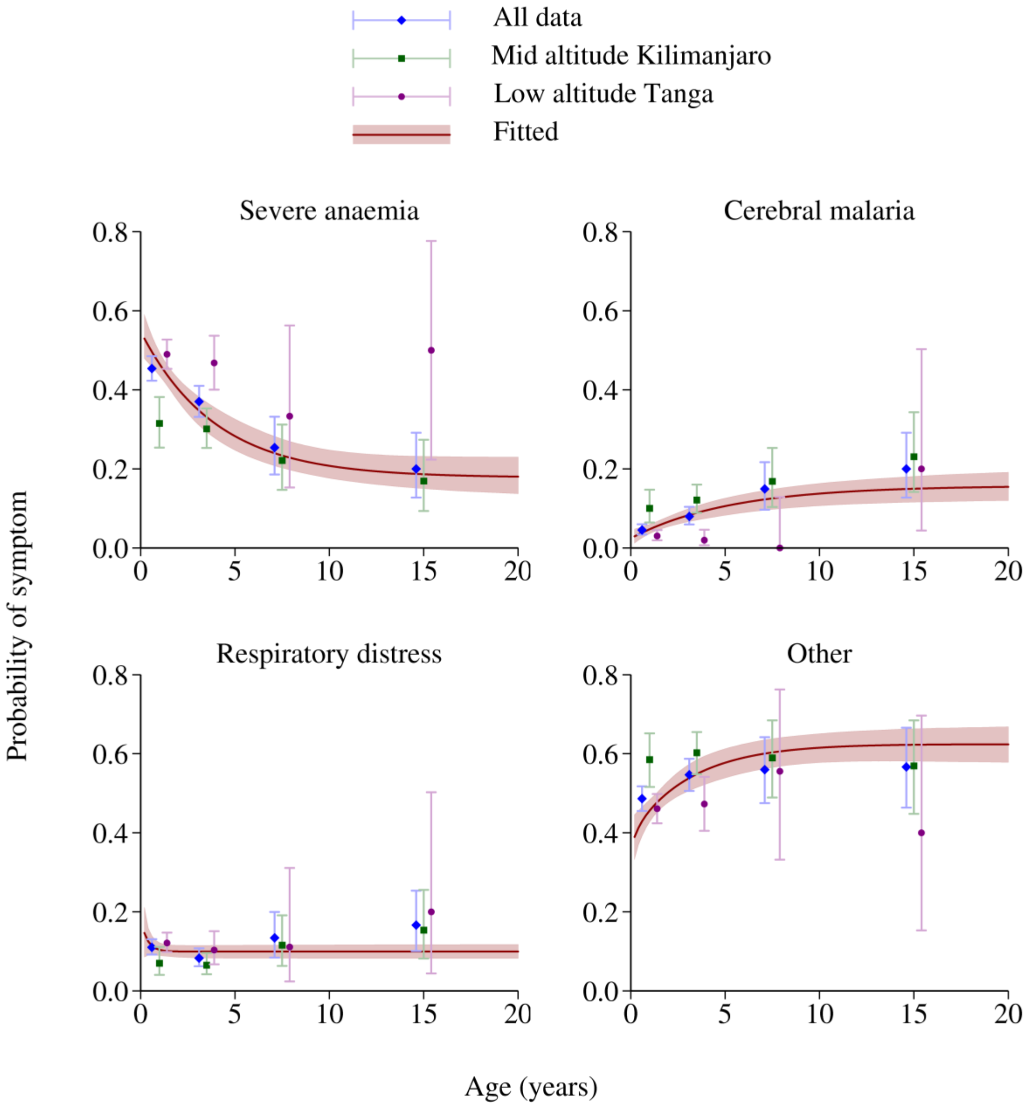

Case fatality by age and transmission intensity

Figure 3D in the main text shows the observed and model predicted case fatality in the Tanzanian dataset. We again plot the observed data for mid altitude Kilimanjaro and low altitude Tanga, in Figure S4. In the fitted model case fatality is assumed to depend only on the symptoms suffered, and so for a given age is assumed to be independent of transmission intensity. There possibly a lower case fatality in the youngest children in mid altitude Kilimanjaro.

Figure S4 Fitted case fatality, and observed data in two areas of Tanzania.

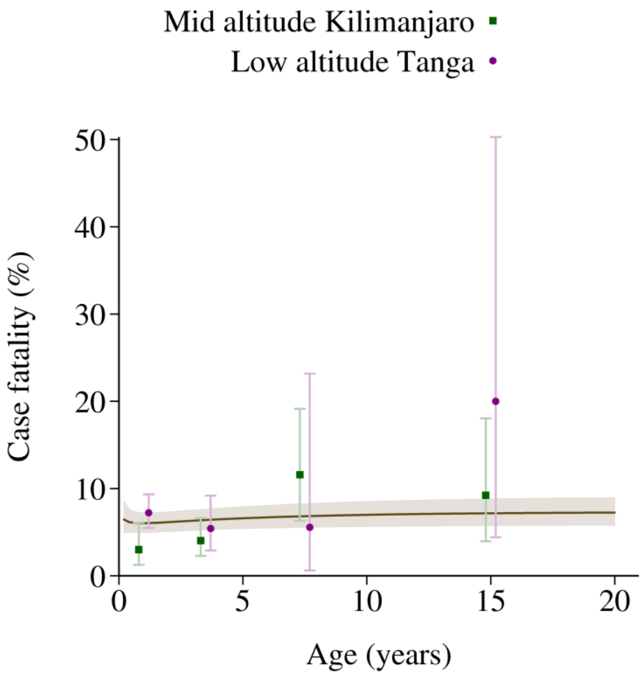

References

1. Reyburn H., Mbatia R., Drakeley C., Bruce J., Carneiro I., Olomi R., Cox J., Nkya W.M., Lemnge M., Greenwood B.M., et al. 2005 Association of transmission intensity and age with clinical manifestations and case fatality of severe Plasmodium falciparum malaria. *JAMA* **293**(12), 1461-1470.

2. Griffin J.T., Ferguson N.M., Ghani A.C. 2014 Estimates of the changing age-burden of Plasmodium falciparum malaria disease in sub-Saharan Africa. *Nat Commun* **5**. (doi:10.1038/ncomms4136).

3. Marsh K., Snow R.W. 1999 Malaria transmission and morbidity. *Parassitologia (Rome)* **41**(1-3), 241-246.

4. Okiro E.A., Al-Taiar A., Reyburn H., Idro R., Berkley J.A., Snow R.W. 2009 Age patterns of severe paediatric malaria and their relationship to Plasmodium falciparum transmission intensity. *Malar J* **8**. (doi:10.1186/1475-2875-8-4).

5. Snow R.W., Omumbo J.A., Lowe B., Molyneux C.S., Obiero J.O., Palmer A., Weber M.W., Pinder M., Nahlen B., Obonyo C., et al. 1997 Relation between severe malaria morbidity in children and level of Plasmodium falciparum transmission in Africa. *Lancet* **349**(9066), 1650-1654. (doi:10.1016/s0140-6736(97)02038-2).

6. Cameron A.C., Trivedi P. 1998 *Regression analysis of count data*. 1 ed, Cambridge University Press.

7. Hay S. 2000 Annual Plasmodium falciparum entomological inoculation rates (EIR) across Africa: literature survey, internet access and review. *Transactions of the Royal Society of Tropical Medicine and Hygiene* **94**(2), 113-127.

8. Rao V.B., Schellenberg D., Ghani A.C. 2013 Overcoming health systems barriers to successful malaria treatment. *Trends Parasitol* **29**(4), 164-180. (doi:10.1016/j.pt.2013.01.005).
